# Flexible ureteroscopy via ureterostomy for managing kidney stones in patients with urinary diversion: a case report

**DOI:** 10.11604/pamj.2025.50.15.46144

**Published:** 2025-01-08

**Authors:** Moussaab Rachid, Ghassane El Omri, Younes Houry, Abdeljalil Heddat

**Affiliations:** 1 Department of Urology, Cheikh Khalifa International University Hospital, Mohammed VI University of Sciences and Health (UM6SS), Casablanca, Morocco

**Keywords:** Flexible ureteroscopy, urinary diversion, cystectomy, urolithiasis, case report

## Abstract

Urinary calculi in patients with urinary diversion, such as bilateral ureterostomies following total cystectomy, present unique challenges due to altered anatomy and physiology. This case highlights the successful use of flexible ureteroscopy via ureterostomy as an alternative approach for managing ureteral stones in this population, adding to the growing body of evidence supporting its feasibility in complex scenarios. A 48-year-old patient presented with sudden-onset right lumbar pain radiating to the external genitalia, consistent with right renal colic, and macroscopic hematuria. Diagnostic imaging revealed a 6 mm obstructing stone in the right kidney associated with ureterohydronephrosis, and laboratory tests showed hyperleukocytosis, elevated C-reactive protein (CRP), and creatinemia. After consultation with the urology team, the patient underwent flexible ureteroscopy through the ureterostomy with in situ laser lithotripsy and placement of a double-J catheter for postoperative drainage. The intervention resulted in complete stone fragmentation, resolution of symptoms, and normalization of renal function. This case underscores the importance of individualized treatment planning and demonstrates that flexible ureteroscopy can serve as a safe and effective minimally invasive alternative to more invasive procedures like percutaneous nephrolithotomy (PCNL) in select patients with altered urinary anatomy.

## Introduction

Urinary calculi are a common complication in patients with urinary diversions, such as bilateral ureterostomies, often necessitating specialized management due to anatomical and physiological alterations [1]. While percutaneous nephrolithotomy (PCNL) remains the gold standard for managing large or complex stones, alternative minimally invasive approaches may be required in certain clinical scenarios.

Flexible ureteroscopy via a ureterostomy represents one such alternative, offering a means to access and treat obstructing stones effectively by utilizing advancements in endourological techniques [2].

In this article, we present the case of a patient with a history of muscle-invasive bladder cancer (MIBC) treated with total cystectomy and bilateral ureterostomy, who subsequently developed urolithiasis and was successfully treated with flexible ureteroscopy through the ureterostomy.

## Patient and observation

**Patient information:** a 48-year-old patient presented to our emergency department with a sudden onset of right lumbar pain. He had a medical history of muscle-invasive bladder cancer (MIBC), for which he had undergone a total cystectomy with bilateral ureterostomy 11 years prior to his admission.

**Clinical findings:** upon admission, the patient reported severe right lumbar pain radiating to the external genitalia, consistent with right renal colic, accompanied by macroscopic hematuria. Clinical examination revealed a conscious patient who was hemodynamically and respiratorily stable, with no evidence of fever. Examination of the abdomen and bilateral ureterostomies did not demonstrate any notable abnormalities.

**Timeline:** eleven years before, the patient was treated at another hospital for a muscle-infiltrating bladder tumor and underwent a total cystectomy with bilateral ureterostomy, combined with neoadjuvant chemotherapy. Since the operation, the patient had reported a favorable outcome with regular follow-up. He had never experienced low back pain until six hours prior to admission, when he developed the sudden onset of intense low back pain associated with macroscopic hematuria, prompting him to seek care at the emergency department of our hospital.

**Figure 1:**
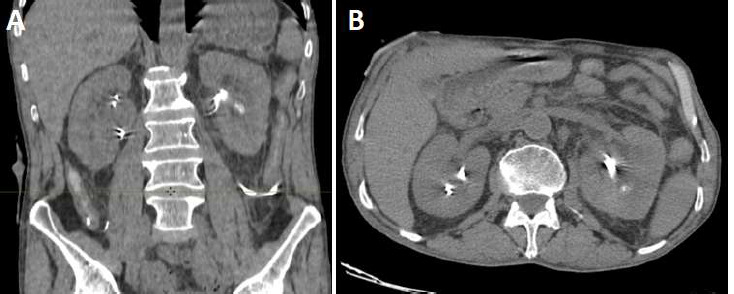
(A, B) bilateral kidney stones with right pyelocaliceal dilatation and calcification in the proximal left ureterostomy

**Diagnostic assessment:** in view of the renal colic syndrome, an abdominopelvic CT scan was performed, revealing an acute obstructive urinary syndrome with ureterohydronephrosis and pyelocaliceal dilatation upstream of a 6 mm pyelic calculus (Figure 1), along with additional bilateral calculi measuring 2 to 6 mm. No abnormalities were observed at the cystectomy site or in the contralateral kidney, except for proximal calcification of the left ureterostomy catheter. Biological tests revealed hyperleukocytosis at 12,500/mm^3^, a CRP level of 140 mg/L, creatinemia of 22 mg/L, and leukocyturia and hematuria on urine cytobacteriological examination (UCE).

**Figure 2:**
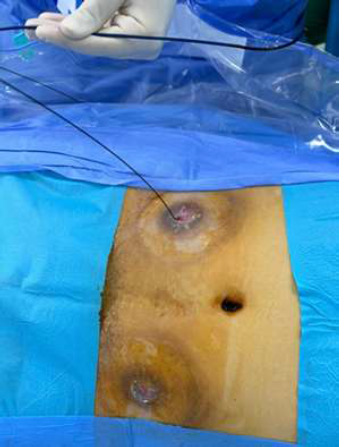
introduction of a hydrophilic safety guide into the right ureter

**Figure 3:**
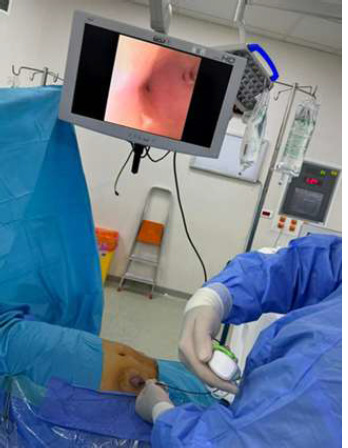
exploration of the right kidney by flexible ureteroscopy through the ureterostomy

**Therapeutic interventions:** after consultation with the urology team, we opted to perform a flexible ureteroscopy through the ureterostomy. The patient was positioned supine under antibiotic prophylaxis and general anesthesia, with the surgeon positioned on the patient's right. The procedure began with the placement of a hydrophilic guidewire in the right ureter (Figure 2). The surgeon then introduced the flexible ureteroscope through the hydrophilic guidewire at the right ureteral level to access the right pyelo-ureteral junction (Figure 3). Once the stones were located, the procedure continued with in situ laser lithotripsy, achieving complete fragmentation of the stones, followed by the insertion of a double-J catheter for postoperative urinary drainage.

**Follow-up and outcome of interventions:** the patient was discharged two days after the operation. The patient attended follow-up consultations one week and one month after the operation, during which he was found to have an uneventful postoperative recovery. His symptoms and hydronephrosis had resolved, and his creatinemia levels were normal.

**Patient perspective:** it was a very stressful experience, especially considering my surgical history. However, I am grateful for the choice of a less invasive approach, which led to a rapid and uncomplicated resolution of my problem. I would like to thank the urology team for their care.

**Informed consent:** it was obtained from the patient for the publication of this article.

## Discussion

The management of urinary calculi in patients with urinary diversion, such as bilateral ureterostomies following total cystectomy, presents unique challenges due to altered anatomy, metabolic changes, and an increased risk of infectious complications [3]. In our case, the decision to use flexible ureteroscopy via the ureterostomy allowed for a minimally invasive approach that proved both safe and effective.

Patients with urinary diversions are predisposed to stone formation due to multiple factors. Metabolically, these patients often experience chronic hyperchloremic metabolic acidosis, increased urinary calcium excretion, and hypocitraturia, all of which contribute to the formation of calcium and infection-related stones. Anatomical factors, such as urine stasis and the presence of foreign bodies (e.g. non-absorbable sutures or stents), further exacerbate the risk [4]. Our patient, with an obstructing 6 mm pyelic stone, demonstrated acute obstructive urinary syndrome, consistent with these known risk factors.

Several studies highlight the challenges of managing urolithiasis in patients with urinary diversions. Percutaneous nephrolithotomy (PCNL) is widely recognized as the gold standard for treating large or complex stones in this population due to its high stone-free rates and proven efficacy [1,5]. However, in select situations where PCNL may not be the most suitable approach, alternative methods such as flexible ureteroscopy can provide effective and minimally invasive solutions [2,6]. In this case, flexible ureteroscopy via the ureterostomy allowed for successful stone management, demonstrating its feasibility as an alternative when anatomical or clinical factors favor less invasive approaches.

Flexible ureteroscopy, although less commonly utilized in patients with urinary diversions, has gained traction with advances in instrumentation [7]. As demonstrated in our case, it offers a minimally invasive alternative for managing ureteral stones, particularly when direct access to the upper urinary tract is achievable. Despite historical concerns about low stone-free rates with ureteroscopy, recent improvements in technique and equipment have yielded better outcomes [8]. In our patient, in situ, laser lithotripsy achieved complete fragmentation of the stone, with no postoperative complications and resolution of symptoms.

The success of this case underscores the importance of individualized treatment planning in patients with urinary diversions [4,9]. While PCNL and shock wave lithotripsy (SWL) remain valuable options, ureteroscopy can serve as a viable alternative, especially in anatomically challenging cases. Furthermore, the case highlights the need for long-term follow-up to monitor for recurrence, as the literature reports recurrence rates as high as 30-50% in this population [10].

Thus, this case demonstrates the feasibility and efficacy of flexible ureteroscopy through a ureterostomy for treating urinary stones in patients with urinary diversion. It also underscores the need for a multidisciplinary approach, integrating advanced endourological techniques and careful perioperative management to optimize outcomes in this complex patient population.

## Conclusion

This case demonstrates the successful use of flexible ureteroscopy via a ureterostomy for managing a symptomatic ureteral stone in a patient with urinary diversion. While PCNL remains the standard of care for complex cases, this approach offers a safe and effective alternative in select circumstances, particularly when anatomical or clinical factors limit other options. The favorable outcome observed reinforces the importance of individualized treatment planning and underscores the role of minimally invasive techniques in the management of urolithiasis in patients with altered urinary anatomy. Further studies are warranted to evaluate the broader applicability of this approach.
